# Serious Adverse Events with Cyanoacrylate Closure of Varicose Veins: An Initial Report from a Large-Scale National Survey in Japan

**DOI:** 10.3400/avd.oa.23-00106

**Published:** 2023-12-08

**Authors:** Michihisa Umetsu, Masayuki Hirokawa, Eri Fukaya, Eiichi Teshima, Hitoshi Kusagawa, Toshiya Nishibe, Hiroko Nemoto, Makoto Mo, Tomohiro Ogawa

**Affiliations:** 1 Division of Vascular Surgery, Department of Surgery, Tohoku University Hospital, Sendai, Miyagi, Japan; 2 Ochanomizu Vascular and Vein Clinic, Tokyo, Japan; 3 Division of Vascular Surgery, Stanford University School of Medicine, Palo Alto, CA, USA; 4 Vascular and Endovascular Surgery, Fukuoka Wajiro Hospital, Fukuoka, Fukuoka, Japan; 5 Matsusaka Ohta Clinic, Matsusaka, Mie, Japan; 6 Department of Medical Management and Informatics, Hokkaido Information University, Ebetsu, Hokkaido, Japan; 7 Department of Surgery, Yokohama City University, Yokohama, Kanagawa, Japan; 8 Department of Cardiovascular Surgery, Yokohama Minami Kyosai Hospital, Yokohama, Kanagawa, Japan; 9 Japanese Society of Phlebology, Tokyo, Japan; 10 Department of Cardiovascular Surgery, Fukushima Dai-Ichi Hospital, Fukushima, Fukushima, Japan; 11 Japanese Regulatory Committee for Endovascular Treatment of Varicose Veins, Tokyo, Japan

**Keywords:** adverse event, complication, cyanoacrylate closure, national survey, varicose vein

## Abstract

**Objective:** Cyanoacrylate closure (CAC) is a minimally invasive technique for the treatment of varicose veins. A recent paper reported serious adverse events (AEs) associated with this use. This triggered an urgent survey to determine the incidence of AEs in Japan.

**Methods:** The CAC-AE survey was sent to all 1,030 institutions authorized for CAC treatments. Cases performed between January 2020 and October 2023 were surveyed. Data on serious AEs and mortality were collected.

**Results:** There were 623 surveys returned. There were 16 cases of proximal deep vein thrombosis, 3 cases of pulmonary embolism (PE), and 0 cases of stroke. Deep vein occlusion due to cyanoacrylate extension was observed in 1 case. Vein resection due to infection was observed in 4 cases. There were 299 cases of localized phlebitis and/or allergic reactions requiring steroid administration. Systemic allergic reactions requiring steroid administration were observed in 66 cases. There was no anaphylaxis associated with cyanoacrylate. There was one postoperative death from PE.

**Conclusion:** This report’s intent is to provide real world data on serious AEs following CAC from Japan given current concern over these events. An extensive report investigation of individual complications with analysis including causality will be provided following a full investigation separately.

## Introduction

Cyanoacrylate closure (CAC) for varicose veins is a nonthermal, nontumescent treatment described as safe and minimally invasive for varicose veins, and many clinical studies have reported that serious complications of CAC are rare.^1)^ The cyanoacrylate used in CAC is used in intra-arterial catheter embolization and wound closure and has a long history of safety although it also has been known to be an allergen.^2)^ Parsi et al. recently reported large numbers of serious adverse events (AEs), including death and stroke, from event databases in the United States, Australia, and the United Kingdom.^3)^ Less morbid AEs including phlebitis have been reported in 11%–15% of patients with CAC,^4^^–^^6)^ some associated with type I and type IV hypersensitivity. Additionally, there are concerns regarding the long-term effects on the immune system. Foreign body granulomas (FBGs) are rare, but it may require resection of the treated vein for resolution and is due to an inflammatory immune response. Although these serious AEs associated with CAC have been reported both as case reports and in a systematic review, their incidence is difficult to discern as the case denominator is unknown.^7^^,^^8)^

In Japan, certification by the Japanese Regulatory Committee for Endovascular Treatment of Varicose Veins (JRCETVV) is required to perform VenaSeal as there is a mandatory requirement by the Pharmaceuticals and Medical Device Agency (PMDA) to collect post marketing safety information including AEs for newly available medical devices. VenaSeal is the only cyanoacrylate treatment approved in the venous space. Other cyanoacrylate products used in Japan are not regulatory cleared. VenaSeal became available and a covered procedure under the national health insurance in December 2019.^6^^,^^9)^ An estimated 33000 CAC treatments in 476 institutions have been performed based on VenaSeal shipment records provided by Covidien Japan.

This CAC-AE survey was urgently performed nationwide to understand the incidence of serious AEs in Japan in CAC treatment by JRCETVV.

## Materials and Methods

### Study design and study population

The CAC-AE survey was administered as a web-based questionnaire between November 7 and 17, 2023, for physicians and institutions registered with JRCETVV. Data were collected on patients who had completed their procedures on or before October 31, 2023. Reports of serious AEs were thoroughly vetted including data verification to confirm the events were real.

We determined serious AEs as follows: proximal deep vein thrombosis (DVT), pulmonary embolism (PE), stroke, deep vein occlusion by cyanoacrylate extension, serious infection, anaphylaxis, granuloma, major bleeding, localized phlebitis requiring systemic steroid administration, and systemic hypersensitivity. Serious outcomes were determined as death and need for vein resection. Deep vein occlusion by cyanoacrylate extension was defined as deep vein occlusion due to injected glue flowing into a deep vein or accidental catheter insertion into a deep vein. Serious infection was defined as cases of requiring systemic antibiotics and hospitalization due to sepsis or vein resection. FBG was defined as a lesion of redness, swelling, and induration of granuloma in the cyanoacrylate-treated area several months to years after CAC, including those resulting in skin eruption. Serious AEs related to localized phlebitis and systemic hypersensitivity were determined as cases of requiring oral and/or intravenous steroids.

### Ethical issues

This study received approval from the institutional review board of Fukuoka Wajiro Hospital (approval number: 00220, approval date: November 13, 2023). This survey is concordant with the ethical guidelines for medical and biological research involving human subjects issued by the Ministry of Health, Labor, and Welfare in Japan.

## Results

A total of 623 (60.5%) medical institutions responded to the survey. Of these, 328 (52.6%) institutions performed CAC treatments. The responses were reviewed by three independent physicians of the JRCETVV.

**Table 1** shows serious AEs and outcomes after CAC. There were 16 cases of proximal DVT, 3 cases of PE, and 1 case of deep vein occlusion with cyanoacrylate extension. There was no anaphylaxis associated with cyanoacrylate. There were 299 cases of localized phlebitis and 66 cases of systemic hypersensitivity requiring oral or intravenous steroids. There were a total of 5 cases requiring vein resection: 4 were due to infection and 1 was a case of FBG. There were no strokes, granulomas causing skin ulceration, or major bleeding complications.

**Table:** Table 1 Serious adverse events and outcomes of CAC

Adverse events	n
Proximal DVT	16
Pulmonary thromboembolism	3
Stroke	0
Deep vein occlusion by cyanoacrylate extension	1
Serious infection requiring glue resection	4
Granuloma	1
Major bleeding	0
Anaphylaxis	0
Localized phlebitis requiring systemic steroids	299
Systemic allergy requiring systemic steroids	66
Outcomes	
Death	1
Glue resection	5

CAC: cyanoacrylate closure; DVT: deep vein thrombosis

There was 1 case of death due to PE in a woman in her 80s, 6 weeks after CAC under general anesthesia. There were no other serious AE concerns collected from the survey.

## Discussion

In Japan, CAC is a PMDA-approved treatment covered by national health insurance in 2019, and physicians must be certified by JRCETVV to perform CAC. The JRCETVV organizes and manages CAC education and certifications for practice based on regulatory requirements by the PMDA. The JRCETVV consists of members from the Japanese Society of Phlebology, the Japanese Society of Vascular Surgery, the Japanese Society of Angiology, the Japan Society of Plastic and Reconstructive Surgery, the Japanese Dermatological Association, and the Japanese Society for Interventional Radiology. The JRCETVV previously conducted a nationwide survey of venous thromboembolic complications immediately after introducing endovenous ablation treatments to examine their safety.^10)^ Prior to the widespread use of CAC, a Japanese CAC guideline was published to provide knowledge of complications and share indications.^6)^ Noted in the guideline was the risk of infection and immune reaction given that the injected cyanoacrylate will remain in the body as a foreign body indefinitely. The guideline clearly noted that CAC should not be performed in venous leg ulcers complicated by infection. CAC is also relatively contraindicated in patients predisposed to allergic reactions or with a history of granulomatous disease.

According to the Food and Drug Administration’s (FDA’s) Manufacturer and User Facility Device Experience (MAUDE) Adverse Event Report, the possible cause of death after CAC treatment was sepsis or PE. One death was identified in the current study, and the cause of death was also PE. In this case, the patient underwent CAC under general anesthesia and developed bilateral DVT and PE 10*–*15 days following the procedure and died at 6 weeks. We could not verify whether this AE was due to CAC or if the patient had other risk factors. By comparison, in a previous report of 43203 cases after endovenous laser ablation (EVLA) in Japan, the incidence of PE (3 cases) and endovenous heat-induced thrombus class 4 (7) were reported to be 0.0067% and 0.013%, respectively.^10)^

In the MAUDE report, all deaths due to infection were in cases of CAC treatments performed on venous leg ulcers. In our survey, there were no deaths due to sepsis, but there were 4 cases of vein resection due to infection.^8)^

No anaphylaxis was noted in this survey. However, 299 patients with phlebitis and/or a delayed hypersensitivity reaction required systemic steroids. Among the patients with systemic allergy, 66 patients required systemic steroids. Non-life-threatening AEs specific to CAC including glue-related complications at the puncture site and persistent hyperpigmentation in the area of the injection were also observed.^11)^

CAC is a minimally invasive method of treating axial vein reflux. However, there are several AEs that differ from other methods of endovenous ablation, including the need for vein/glue resection due to infection and hypersensitivity reactions.

## Conclusion

These initial data from the CAC-AE survey as a rapid communication provide accurate validated data amid concerns and confusion in clinical practice using CAC. This urgent nationwide survey found several serious AEs of CAC in Japan. A complete data analysis and detailed investigation of individual serious and non-serious AEs will be reported in due course. Further investigation into the AEs will clarify which patients should avoid CAC treatments and will make it a safer treatment with fewer complications.

*(It is important to emphasize that this is composed of preliminary data from institutions that responded to the CAC-AE survey between November 10, 2023 and November 17, 2023. Additional data and detailed information will become available as the JRCETVV efforts on data collection continue.)*

## Acknowledgments

We would like to thank all the physicians who participated in the surveys. We thank Ms. Maeno of the secretariat of JRCETVV for assistance.

## Disclosure Statement

MH is a consultant with Integral Corporation. All other authors have no conflict of interest.

## Author Contributions

Study conception: MM

Data collection: MU, MH, MM, and TO

Analysis: MU

Investigation: MU, MH, MM, and TO

Manuscript preparation: MU, TN, MH, and EF

Funding acquisition: not applicable

Critical review and revision: all authors

Final approval of the article: all authors

Accountability for all aspects of the work: all authors.
